# Genetic characterization of measles virus strains isolated during an epidemic cluster in Puglia, Italy 2006–2007

**DOI:** 10.1186/1743-422X-4-90

**Published:** 2007-09-21

**Authors:** Maria Chironna, Rosa Prato, Anna Sallustio, Domenico Martinelli, Cinzia Germinario, Pierluigi Lopalco, Michele Quarto

**Affiliations:** 1 Hygiene Section, Department of Biomedical Sciences and Human Oncology, University of Bari, Policlinico Piazza G. Cesare 11, 70124 Bari, Italy; 2 Dipartimento di Scienze Mediche e del Lavoro, Hygiene Section, University of Foggia, Italy; 3 Osservatorio Epidemiologico Regione Puglia, Coordinating Centre for Notifiable Diseases, Bari, Italy; 4 European Centre for Disease Prevention and Control, Stockholm, Sweden

## Abstract

The genetic characterization of wild-type measles strains isolated during an epidemic cluster of measles occurred in Puglia (South Italy), between November 2006 and January 2007, was performed. Measles virus (MV) detection was carried out by a nested RT-PCR on 8 of 18 total cases. The viruses were analyzed using the standard genotyping protocols. The N gene sequences of the strains from outbreak were identical to each other, and sequence analysis revealed that the viruses belonged to genotype B3, subgroup B3.1, never identified before in Italy. An importation of measles B3.1 strains from Africa was hypothesized. Molecular surveillance will help to monitor the progress in measles elimination.

## Findings

Measles, a highly contagious exanthematic disease, is still a threat for children both in developing and developed countries despite the introduction of routine vaccination. World Health Organization (WHO) has strengthened the strategies for the control of measles and its related complications and mortality worldwide [1].

Active epidemiologic surveillance, laboratory confirmation of suspected cases and monitoring of measles virus genotypes are also primary goals for countries of the WHO European region involved in the process of eliminating measles [2]. Therefore, molecular epidemiology of measles virus has proven a useful tool for epidemiologic investigation and enables the identification of source of infection and transmission pathways [3].

Measles virus (MV), a RNA virus belonging to Morbillivirus genus of the family of Paramixoviridae, is considered monotypic although genetic heterogeneity has been evidenced among wild strains [4]. Actually, 23 genotypes (A, B1–B3, C1–C2, D1–D10, E, F, G1–G3 and H1–H2) have been recognized circulating in different parts of the world, five of them (B1, D1, E, F, and G1) considered inactive since they have not been detected in the past 15 years [5].

In Italy, a National Elimination Plan for Measles and Congenital Rubella was implemented in 2003 with the aim of elimination of indigenous transmission of measles and of reduction of congenital rubella cases to <1-case/100,000 live births according to WHO program for European Region [6].

In order to reach the objective, laboratory surveillance of cases and identification of contacts during outbreaks and their prompt immunization was set up to limit the spread of infection. In fact, based on the current coverage levels for MMR (measles, mumps and rubella) in Italy (a national average of 88% with the first dose within 24 months of age and 46% with the second dose in 2005) and considering the scenario prospected by mathematical models, periodical outbreaks of the diseases could occur due to the presence of susceptibles [7,8].

Between November 2006 and January 2007 an epidemic cluster of measles was identified in Barletta city, Puglia region (South Italy), among children and teenagers <16 years. We investigated the outbreak by serological and molecular methods and determined the measles genotype involved by sequence-based methods.

At the beginning of January 2006 the Coordinating Center of Notifiable Infectious Diseases (Osservatorio Epidemiologico Regione Puglia) received the notification of some cases of measles in the city of Barletta, located about 50 km at North of Bari, Puglia region, South Italy. A case of measles was defines as a case that met the clinical case definition (clinical picture compatible with measles, i.e., a generalized rash lasting more than three days and a temperature >38°C), as well as one or more of the following symptoms: cough, coryza, Koplik's spots, conjunctivitis. A case of measles was also defined as a case that was laboratory confirmed (anti-measles virus IgM positive or RT-PCR/nested-PCR positive) or as a case that met the clinical case definition and was epidemiologically linked to a laboratory confirmed case. From 19 November 2006 to 9 January 2007, 18 cases of measles were reported, 17 in the city of Barletta and 1 in the city of Monopoli, a city located at South of Bari (about 100 Km distant from Barletta). The case of Monopoli was epidemiologically linked to the cluster of Barletta. The first reported case was a male six-year old boy who had never been vaccinated and who was admitted to hospital with fever >38°C, rhinitis, conjunctivitis and cough on 22 November and who developed rash on 24 November. On 24 November a classmate of the first case presented the same symptoms but was not hospitalized. Two days before hospitalization, the first reported case spent some time together with a male cousin 11 years old, not vaccinated, who developed symptoms (fever, cough and rhinitis) starting from 27 November. He revealed the solely case of measles in the city of Monopoli and was not hospitalized. No clinical samples were available from these three cases for laboratory confirmation of measles. In response to the first notification of measles, an active surveillance was set up to trace all possible cases and susceptible contacts.

**Figure 1:**
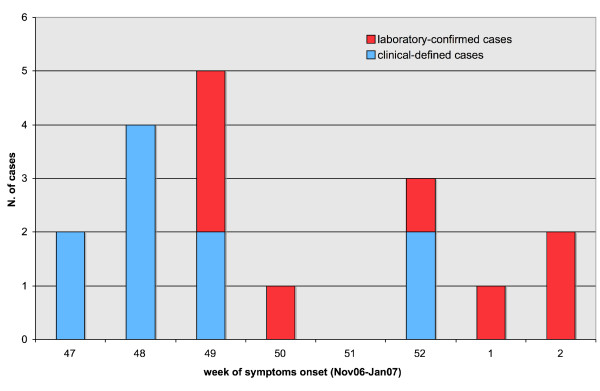
No. of clinical and laboratory-confirmed measles cases by week on onset of symptoms in Puglia region, November 2006–January 2007.

Seven cases of the epidemic cluster were unvaccinated schoolmates of the first reported case. The other 11 unvaccinated cases resided in the same neighborhood. The epidemic curve for the outbreak shows the number of total and laboratory-confirmed measles cases (Figure 1). The 50% of cases were hospitalized. The mean age of children was 6 years old (range: 9 months–15 years). Twenty-eight percent of cases (5) were young children less than 24 months, 33% (6) children aged 3–6 years and 39% (7) children aged 5–15 years. The 55% of subjects were males and the 45 % were females.

After receiving the first notification of measles an extraordinary vaccination campaign for MMR was undertaken and all contacts and susceptible children who attended the school of the town were traced and vaccinated with a first dose of MMR or a second dose if they had previously received only one dose. As a result, 1251 children 2–10 years of age were vaccinated between 12 January and 1 March 2007.

Clinical samples were collected from 12 subjects. Pharyngeal swabs and acute serum samples were obtained from 8 patients and were collected within 2 week after clinical symptoms onset. Convalescent serum samples were obtained for 8 patients. Only for 4 patients both acute and convalescent samples were available.

Sera were tested for the presence of MVs specific IgM by using μ-chain capture Elisa and for specific IgG by indirect Elisa (Measles virus IgM and Measles virus IgG, DiaSorin, Saluggia, Italy).

For direct detection of measles virus, total nucleic acids were extracted from throat swabs by the use of a commercial kit and according to kit manufacturers' (High Pure Viral Nucleic Acid, Roche Diagnostics, Milan, Italy).

The one step cMasterRTplusPCR System (Eppendorf, Hamburg, Germany) was used for retrotranscription and first round PCR. The primers used were Sar1F and Sar1R [9]. A nested PCR was performed on first round negative samples with the Platinum Taq DNA Polymerase High Fidelity (Invitrogen, srl Milan, Italy) and with the primers Sar2F e Sar2R [9]. RNA extracted by 200 μl of a reconstituted dose of MMR vaccine (Priorix, GlaxoSmithKline S.p.A.) was used as positive control for measles detection. Positive samples showed a 444 bp or 229 bp fragment (nested PCR) after agarose gel electrophoresis and UV light visualization.

The 456-bp segment of the N gene of these MV strains, usually used for genotyping, was amplified with the primer MV-N1 and MV-N2 [10].

PCR products were purified using the QIAquick Purification kit (Qiagen S.p.A, Italy) and subjected to sequencing (ABI Prism 3100; Applied Biosystems, Foster City, CA). The relatedness of 456-bp segments of the N gene of the 8 MV strains was assessed through multiple sequence alignment by using ClustalX program. The phylogenetic tree was generated by neighbor-joining method. The output graphics of the tree was produced with NJplot package.

Measles was laboratory confirmed in 8 patients of the outbreak. All these patients resulted positive by molecular detection (3 after first round PCR and 5 after nested-PCR). Seven acute serum samples resulted positive for measles IgM antibodies. In one case measles was laboratory confirmed only by RT-PCR on pharyngeal swab since the serum sample was negative for specific IgM and IgG. Of the 7 IgM positives sera, 4 resulted negative for IgG whereas in 3 cases a contemporary presence of measles specific IgM and IgG antibodies was detected. Four patients of the epidemic cluster clinically diagnosed as measles cases and whose convalescent sera were available, showed positivity solely for IgG antibodies.

**Figure 2:**
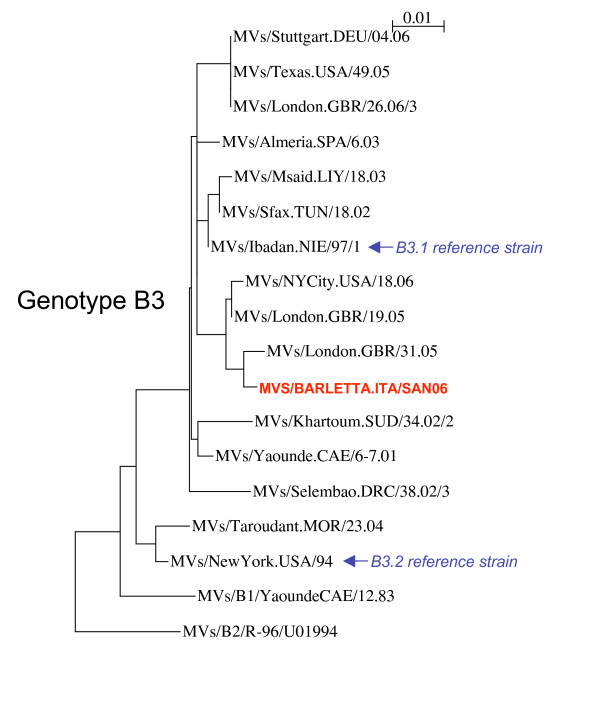
Phylogenetic comparison of the N gene sequences of a wild-type B3.1 reference sequence (MVs/Barletta.ITA/San06) isolated from Puglia during the outbreak and other previously described genotype B3 strains. The scale bar indicates 1% sequence diversity. Following are the GenBank accession numbers of B3 strains used for sequence analysis. MVs/Stuttgart.DEU/04.06: DQ665363; MVs/Texas.USA/49.05: DQ888750; MVs/London.GBR/26.06/3: EF079148; MVs/Almeria.SPA/6.03: AY551544; MVs/Msaid.LIY/18.03: AJ783817; MVs/Sfax.TUN/18.02: AJ783818; MVs/Ibadan.NIE/97/1: AJ232212; MVs/NYCity.USA/18.06: DQ888753; MVs/London.GBR/19.05: EF079127; MVs/London.GBR/31.05: EF079129; MVs/Khartoum.SUD/34.02/2: AY456407; MVs/Yaounde.CAE/6-7.01: DQ267518; MVs/Selembao.DRC/38.02/3: AY274610; MVs/Taroudant.MOR/23.04: DQ779216; MVs/NewYork.USA/94: L46753.

Sequence analysis revealed a complete nucleotide identity among strains in the N gene and genotyping showed a genotype B3 (subgroup B3.1) (Figure 2). This is the first report of the detection of genotype B3 in Italy. The strains characterized showed the highest degree of identity (99%) with the strain MVs/London.GBR/31.05 circulating in UK in 2005 (GenBank accession number EF079129). The molecular characterization of the strains revealed an apparently imported MVs strains although the origin of outbreak was not known despite an accurate epidemiologic investigation. Genotype B3 has been the most frequently detected measles genotype in western and central Africa [10,11]. Several importations of B3 strains into Europe and USA have recently been reported by Rota et al. [12]. Nevertheless, the B3 strains characterized during the current outbreak did not show strict relationships with the strains reported in the latter study. On the other hand, the geographical position of Italy in the Mediterranean basin and the continuous influx of refugees also from sub-Saharan Africa could have enhanced the importation of such measles strains.

In Italy, no specific reports on the molecular epidemiology of measles strains are available. Nevertheless, a review on temporal and geographical distribution of measles genotypes indicated the presence, in Italy, during 1950s–2004 of endemic genotypes C2 (1995), D6 (1996–1997), D8 (1999), and D7 (2002–2003) [13].

More recently, in Italy, two outbreaks of measles have been reported caused by D4 imported measles genotype [14,15]. The first outbreak occurring in Grosseto (southern Tuscany) in 2006 it is likely that originated from an imported case from India where D4 genotype is endemic [7]. The second outbreak occurred in Rome and Lazio region (central Italy), also due to D4 genotype, originated from a nomadic gipsy population (Roma-Sinti) [15].

Importation of measles is now the main cause of possible outbreaks in countries that have eliminated indigenous measles. Therefore, in order to reach the goal set by WHO to eliminate measles from the European region by 2010 [16-18], strengthened active surveillance and molecular characterization of the strains is crucial for monitoring the progress in measles elimination.

A representative sequence reported in this paper (MVs/Barletta.ITA/San06) has been deposited in the GenBank sequence database under Accession number EF468495.

## Competing interests

The author(s) declare that they have no competing interests.

## Authors' contributions

MC and RP analyzed data, interpreted the results and prepared draft manuscript. MC also carried out sequence analysis. AS collected specimens and performed RT/nested PCR, sequence analysis and serologic tests. DM collaborated to data collection and analysis. CG, PL and MQ supervised the study, discussed the results and revised the final draft. All authors read and approved the final manuscript.
